# Prevalence of overactive bladder symptoms and their impact on health-related quality of life of medical and dentistry students: a multicenter cross-sectional study

**DOI:** 10.1186/s12894-021-00909-1

**Published:** 2021-10-08

**Authors:** Ramzi Shawahna, Hatim Hijaz, Khaled Jallad, Mohammad Abushamma, Mothana Sawafta

**Affiliations:** 1 Department of Physiology, Pharmacology and Toxicology, Faculty of Medicine and Health Sciences, An-Najah National University, New Campus, Building: 19, Office: 1340, P.O. Box 7, Nablus, Palestine; 2 An-Najah BioSciences Unit, Centre for Poison Control, Chemical and Biological Analysis, An-Najah National University, Nablus, Palestine; 3 Department of Medicine, Faculty of Medicine and Health Sciences, An-Najah National University, Nablus, Palestine; 4 An-Najah National University Hospital, An-Najah National University, Nablus, Palestine

**Keywords:** Overactive bladder, Medical students, Dentistry students, Health-related quality of life, Palestine

## Abstract

**Background:**

Overactive bladder (OAB) is a popular distressing health condition that has negative impact on health-related quality of life (HRQoL) of the inflicted individuals. This multicenter study was conducted to determine the prevalence of OAB symptoms and their impact on the HRQoL of medical and dentistry students.

**Methods:**

This study was conducted in a cross-sectional design in the 3 main universities in Palestine. In addition to the sociodemographic, health, and academic characteristics of the medical and dentistry students, the questionnaire also contained the OAB symptom bother (6-items) and HRQoL (13-items) Short-Form (OAB-q SF) scales. Kruskal–Wallis test, Mann–Whitney U test, Pearson Chi-Square/Fisher's Exact Test, Spearman’s rank correlations, and a multiple linear regression model were used to analyze the data.

**Results:**

Responses were collected from medical and dentistry students (*n* = 402). The median OAB symptom bother score was 54.1 [44.8, 81.9] and the median HRQoL score was 94.4 [88.4, 94.4]. There was a strong negative correlation between the OAB and HRQoL scores (Spearman’s rho = 64.4%, *p* value < 0.001). OAB scores were significantly higher among dentistry students, females, who had chronic disease, and those who reported stressful life. HRQoL scores were significantly higher among medicine students, those who reported less stressful life, and those who reported satisfaction with their social life. Dentistry students, female, and those who self-reported high stress were 1.94-fold (95% CI 1.05, 3.56), 1.91-fold (95% CI 1.16, 3.14), and 1.88-fold (95% CI 1.21, 2.91) more likely to report less than optimal HRQoL compared to medicine students, male, and those who self-reported low stress, respectively.

**Conclusions:**

Our findings suggested that OAB symptoms were prevalent among medical and dentistry students across Palestinian universities. Decision makers in academia, healthcare authorities, and advocacy groups might need to design appropriate interventions to address health and wellbeing issues of medical and dentistry students. Using appropriate diagnostic procedures, reducing stress, and improving the social life might help in reducing the burden on OAB and improve the HRQoL of medical and dentistry students. More investigations should be conducted to investigate if such interventions are effective in reducing OAB symptoms and improving HRQoL.

**Supplementary Information:**

The online version contains supplementary material available at 10.1186/s12894-021-00909-1.

## Background

Overactive bladder (OAB) is a popular and distressing condition that has negative impact on health-related quality of life (HRQoL) of the inflicted individuals [1]. According to the International Continence Society, OAB is symptomatic health condition characterized by abnormal urinary urgency that is often accompanied by nocturia and/or daytime frequency that could be with or without urgent urinary incontinence in the absence of any obvious urinary tract infections and/or any other underlying pathologies [2, 3]. Previous epidemiological studies have reported variable prevalence rates of OAB among the general public that ranged from 3 to 43% [4–7]. Previous studies also reported that OAB was largely underestimated and undertreated among the general public [7, 8].

Previous studies have reported negative impact of OAB on the HRQoL that can be translated as negative effects on the physical, social, and psychological well-being [9]. OAB can also have negative interference with the daily activities of the affected individuals [9, 10]. Additionally, OAB affects the patients’ relationships with spouse/partner, family, and friends. As a result, anxiety, depression, embarrassment, and difficulties in sleep and sexual relationships were previously reported as common among individuals with OAB [11].

Previous studies have identified different factors that could promote urinary incontinence and OAB like occupational stress, delayed voiding, consumption of tea, carbonated drinks, and smoking [8, 12–14]. The literature reported little on the prevalence of OAB symptoms among young adults and its impact of their HRQoL [8, 13, 14]. Additionally, little was reported on the factors associated with OAB symptoms among young adults [13]. Reisch et al. reported that OAB symptoms were prevalent among 21.7% of female health profession students in a university in the Pacific Northwest [13]. OAB symptoms was more prevalent among participants who consumed caffeine and carbonated drinks compared to those who did not consume caffeine and carbonated drinks. The study also reported lower HRQoL among students with OAB symptoms compared to those without OAB symptoms. Özgür Yeniel et al. OAB symptoms were prevalent among 35.4% of Turkish midwifery students [8]. Turkish midwifery students with OAB symptoms also reported lower HRQoL compared to those without OAB symptoms. Similarly, Hagovska et al. reported lower HRQoL among overweight female university students with OAB symptoms compared to students without OAB symptoms [14].

Studying medicine and dentistry can be highly stressful. Throughout their medical school, medical and dentistry students face multiple highly demanding tasks [15]. As a result, medical and dentistry students often reported lower HRQoL compared to their age-matched peers and students of other disciplines [16–18]. Additionally, previous studies have reported higher prevalence of anxiety and depression symptoms among medical and dentistry students when compared to their age-matched groups of students in other non-medical disciplines [19–21].

Recently, there has been many calls to improve the HRQoL of medical and dentistry students [18, 22, 23]. As OAB symptoms are common and distressing, they could also worsen the HRQoL of medical and dentistry students. Little was reported on the prevalence of OAB symptoms among medical and dentistry students. Additionally, little was reported on the factors that could be associated with prevalence of OAB symptoms and the HRQoL of medical and dentistry students. Associations between sociodemographic, health, and academic variables with OAB symptoms and HRQoL were not investigated. Therefore, this study was conducted to determine the prevalence of OAB symptoms among the medical and dentistry students in Palestine. The study also aimed to investigate the associations between the sociodemographic, health, and academic characteristics of the medical and dentistry students with the prevalence of OAB symptoms and their HRQoL.

## Methods

### Study settings/context

This study was conducted in the 3 major universities in Palestine. The Doctor of Medicine (MD) program in Palestinian universities consists of 265 credited hours of basic and clinical courses/training and the dentistry program consists of 210 credited hours. The students often complete one of the programs in 6 academic years. Graduates are the future physicians and dentists in primary, secondary, and tertiary healthcare centers in Palestine. The study was conducted in the context of improving the HRQoL of the medical and dentistry students in Palestine.

### Study design and the study tool

This study was conducted in a cross-sectional design among medical and dentistry students in Palestine using a questionnaire as the study tool. The study was conducted and reported in compliance with the Strengthening the Reporting of Observational Studies in Epidemiology (STROBE) Statement [24]. Compliance with the STROBE statement is shown in Additional file 1: Table S1. Because the study was conducted during the ongoing COVID-19 pandemic, an online version was used to collect the data in this study.

The questionnaire contained two parts. The first part collected sociodemographic, health, and academic characteristics of the medical and dentistry students like age, gender, height, body weight, smoking habits, employment status, presence of chronic disease, history of surgery, history of trauma, the university where the student was enrolled, academic discipline, academic year, number of study hours, self-rated satisfaction with the academic achievement, self-rated degree of stress, self-rated satisfaction with the financial status, and self-rated satisfaction with the social life. The second part contained the Overactive Bladder Symptom and Health-Related Quality of Life Short-Form (OAB-q SF) [25]. The OAB symptom bother scale contained 6-items and the HRQoL Scale contained 13-items. The Scales were abridged from the 33-items OAB-q Scale [26]. The OAB-q SF measures three HRQoL domains: coping, sleep, and emotional/social interactions. Higher OAB symptom bother scores and lower HRQoL indicate greater effect [25, 26]. The scales were developed and assessed for subscale structure and psychometric performance [25]. The scales were shown to have excellent model fit and internal consistency. The convergent validity, discriminant validity, and internal reliability of the scales were good. Scores of the scales were reproducible and responsive to change.

### Pilot testing of the questionnaire

The questionnaire was pilot tested among 18 medical students who did not take part in the larger study. The students were asked to evaluate the questionnaire for readability and comprehensibility. Based on the feedback of the students in the pilot testing, some items were revised for clarity. To ensure the stability of scores over a short period of time, the test–retest method was used. The students were asked to respond to the questionnaire. After a short time period (30 min to 2 h), the same students who participated in the pilot testing were asked to respond to the questionnaire once again. The scores obtained in both rounds were correlated using Pearson’s correlations. It was decided a priori that a Pearson’s correlation coefficient of ≥ 80% would be considered acceptable to indicate stability of the scores over a short time period. The Cronbach’s alpha statistics were used to assess the relatedness of the items (internal consistency of the study tool). It was decided a priori that a Cronbach’s alpha of ≥ 70% would be considered acceptable to indicate internal consistency of the questionnaire.

### Study population, sample size, and sampling

The study population in this study was medical and dentistry students in Palestine. We used an online sample size calculator to calculate the sample size needed for this study. Assuming a population of approximately 9000 medical and dentistry students in the different Palestinian universities, the sample size calculated at a 95% confidence interval (CI) and accepting a 5% margin of error. The sample size needed for this study was 369 students. Medical and dentistry students in the 3 major universities in Palestine were invited to take part in the study using their formal mailing groups. A convenience sampling technique was used and the data collection was terminated when 402 responses were obtained.

### Data analysis

The data collected were entered into SPSS for Windows v.21.0. Sociodemographic, health, and academic data were grouped into categories. The OAB-q SF consisted of 2 domains: OAB symptom bother (6-items) and HRQoL (13-items). The students could rate each of the OAB symptom bother items on a Likert scale of 1–6 (1 = not at all, 6 = a very great deal). The possible raw scores of the OAB symptom bother items could range from 6 to 36 (possible raw score range = 30). Similarly, the students could rate each of the HRQoL items on a Likert scale of 1–6 (1 = none of the time, 6 = all of the time). The lowest and highest possible raw scores of the HRQoL items could range from 13 to 78 (possible raw score range = 65) [25].

Scores of the OAB symptom bother (6-items) were transformed as in the Eq. (1):

1 $${\text{Transformed Score}} = \frac{{\left( {{\text{Actual raw score }} - {\text{lowest possible raw score}}} \right)}}{{\text{Possible raw score range}}} \times 100{\text{\% }}$$

Scores of the QoL scores were transformed as in the Eq. (2):

2 $${\text{Transformed Score}} = \frac{{\left( {{\text{Highest possible score }} - {\text{Actual raw score}}} \right)}}{{\text{Possible raw score range}}} \times 100{\text{\% }}$$

The transformed scores of the OAB symptom bother and HRQoL items were assessed for normal distribution using Kolmogorov–Smirnov test of normality. As the distribution of the data was not normal, nonparametric tests were used. The data are presented as median [lower quartile (Q1), upper quartile (Q3)]. Categories were compared using Kruskal–Wallis test or Mann–Whitney *U* test, as appropriate. Associations between sociodemographic, health, and academic variables of the medical and dentistry students and presence of OAB symptoms and less than optimal HRQoL were investigated using Pearson Chi-Square (χ^2^)/Fisher's Exact Test (FET). Correlations were investigated using Spearman’s rank correlations. To control potential confounding factors, a multiple linear regression model was used to investigate predictors of higher scores. Then variables with statistically significant differences in the Mann–Whitney *U* test were retained in the regression model. The goodness of fit of the model was indicated by an R^2^ value of 0.30. The variance inflation factor (VIF) and tolerance were assessed using collinearity statistics. Tolerance values were > 1.02 and the VIF values were less than 1.50 which indicated absence of multicollinearity issues. Presence of OAB symptom bother was indicated by a sum transformed score of > 35.6 on the 6-item OAB symptom bother Scale and less than optimal HRQoL was indicated by a sum transformed score of < 94.4 on the 13-item HRQoL Scale. A multivariate logistic regression model was used to determine which variables were predictors of presence of any OAB symptom bother and less than optimal HRQoL. Odds ratios (OR) with their 95% CI were calculated. Statistical significance was considered when the p-value was < 0.05.

### Ethics approval and consent to participate

The study was conducted in compliance with local and international ethical guidelines including those followed at An-Najah National University and Declaration of Helsinki. Because this study was conducted in the context of improving the HRQoL of the medical and dentistry students in Palestine and in view of the no/minimal risk to the study participants, this study fell within the “Exempt” review category. The Institutional Review Board (IRB) of An-Najah National University approved this exemption. All students provided written informed consent to participate in the study.

## Results

### Reliability of the study tool

When the scores of the students in the pilot testing were correlated, the Pearson’s correlation coefficient (r) was 93.0%. This indicated an excellent stability of scores over the short period of time. The Cronbach’s alpha of all items (6 OAB symptom bother and 13 HRQoL items) was 93.3% (95% CI of 92.4% to 94.3%). For the 6 OAB symptom bother items, the Cronbach’s alpha was 81.1% (95% CI of 78.1% to 83.8%). For the 13 HRQoL items, the Cronbach’s alpha was 93.3% (95% CI of 92.3% to 94.2%). This indicated that the items in each domain as well as in the questionnaire as a whole were internally consistent.

### Sociodemographic, health, and academic characteristics of the medical and dentistry students

Responses (*n* = 402) were collected from medical and dentistry students who were enrolled in the 3 major universities in Palestine. Of the students, 276 (68.7%) were female, 205 (51.0%) were in their clinical stage (3rd to 6th year), 228 (56.7%) were 21 years and older, 131 (32.6%) were overweight or obese, 24 (6.0%) had chronic disease, 78 (19.4%) had a history of surgery, 8 (2.0%) had a history of trauma, 14 (3.5%) had a side/part-time job, 65 (16.2%) were smokers, 320 (79.6%) were satisfied with their academic achievement, 147 (36.6%) rated their life as stressful, 225 (56.0%) studied for 5 or more hours per day, 253 (62.9%) were satisfied with their financial status as high, and 277 (68.9%) were satisfied with their social life. Medical and dentistry students were similar with regard to their sociodemographic, health, and academic characteristics except for smoking habits and number of study hours/day. Dentistry students smoked more than medical students (Pearson Chi-Square/Fisher's Exact Test = 5.10, *p* value = 0.028). On the other hand, medical students studied more than dentistry students (Pearson Chi-Square/Fisher's Exact Test = 5.59, *p* value = 0.024). Comparison between the sociodemographic, health, and academic characteristics of the medical and dentistry students is shown in Additional file 1: Table S2. Detailed sociodemographic, health, and academic characteristics of the medical and dentistry students are shown in Table 1.

**Table 1:** Sociodemographic, health, and academic characteristics of the students who participated in the study (*n* = 402)

Variable	n	%
University*		
University 1	250	62.2
University 2	114	28.4
University 3	38	9.5
Discipline		
Medicine	350	87.1
Dentistry	52	12.9
Academic stage		
Basic stage (1st–3rd year)	197	49.0
Clinical stage (4th–6th year)	205	51.0
Gender		
Male	126	31.3
Female	276	68.7
Age (years)		
< 21	174	43.3
≥ 21	228	56.7
Body mass index		
< 24.9	271	67.4
≥ 24.9	131	32.6
Presence of chronic disease		
No	378	94.0
Yes	24	6.0
History of surgery		
Yes	78	19.4
No	324	80.6
History of trauma		
Yes	8	2.0
No	394	98.0
Employment		
Yes	14	3.5
No	388	96.5
Smoking		
Yes	65	16.2
No	337	83.8
Self-rated satisfaction with academic achievement		
Not satisfied	82	20.4
Satisfied	320	79.6
Self-rated stress		
Low stress	255	63.4
High stress	147	36.6
Number of study hours/day		
< 5	177	44.0
≥ 5	225	56.0
Self-rated satisfaction with the financial status		
Not satisfied	149	37.1
Satisfied	253	62.9
Self-rated satisfaction with the social life		
Not satisfied	125	31.1
Satisfied	277	68.9

*1, 2, and 3 denotes the largest universities in Palestine in terms of number of students

### Prevalence of OAB symptom bother among medical and dentistry students

The median transformed OAB symptom bother among medical and dentistry students was 54.1 [44.8, 81.9]. The students reported some OAB symptom bother relevant to an uncomfortable urge to urinate (60.4%), a sudden urge to urinate with little or no warning (36.3%), accidental loss of small amounts of urine (20.1%), nighttime urination (28.1%), waking up at night because you had to urinate (43.8%), and urine loss associated with a strong desire to urinate, respectively (16.9%). Distribution of the rating of the medical and dentistry students on the 6 OAB symptom bother items is shown in Table 2.

**Table 2:** Distribution of the responses of the medical and dentistry students on the 6 overactive bladder symptom bother items (*n* = 402)

#	Item	Not at all		A little bit		Some-what		Quite a bit		A great deal		A very great deal	
		n	%	n	%	n	%	n	%	n	%	n	%
1	An uncomfortable urge to urinate	159	39.6	118	29.4	46	11.4	48	11.9	23	5.7	8	2.0
2	A sudden urge to urinate with little or no warning	256	63.7	75	18.7	29	7.2	31	7.7	8	2.0	3	0.7
3	Accidental loss of small amounts of urine	321	79.9	48	11.9	12	3.0	15	3.7	5	1.2	1	0.2
4	Nighttime urination	289	71.9	71	17.7	14	3.5	16	4.0	8	2.0	4	1.0
5	Waking up at night because you had to urinate	226	56.2	120	29.9	20	5.0	19	4.7	14	3.5	3	0.7
6	Urine loss associated with a strong desire to urinate	334	83.1	37	9.2	16	4.0	7	1.7	8	2.0	0	0.0

### Health related quality of life scores

The median transformed HRQoL scores was 94.4 [88.4, 94.4]. The students reported less than optimal HRQoL scores relevant to the domains: coping, sleep, and emotional/social interactions. The detailed responses of the medical and dentistry students on the 13 HRQoL items are shown in Table 3.

**Table 3:** Distribution of the responses of the medical and dentistry students on the 13 health-related quality of life items (*n* = 402)

#	Item	None of the time		A little of the time		Some of the time		A good bit of the time		Most of the time		All of the time	
		n	%	n	%	n	%	n	%	n	%	n	%
1	Caused you to plan ‘‘escape routes’’ to restrooms in public places	315	78.4	42	10.4	18	4.5	15	3.7	8	2.0	4	1.0
2	Made you feel like there is something wrong with you	327	81.3	37	9.2	9	2.2	14	3.5	9	2.2	6	1.5
3	Interfered with your ability to get a good night’s rest	352	87.6	25	6.2	4	1.0	12	3.0	6	1.5	3	0.7
4	Made you frustrated or annoyed about the amount of time you spend in the restroom	355	88.3	28	7.0	6	1.5	4	1.0	4	1.0	5	1.2
5	Made you avoid activities away from restrooms (i.e., walks, running, hiking)	364	90.5	0	0.0	21	5.2	12	3.0	2	0.5	3	0.7
6	Awakened you during sleep	316	78.6	52	12.9	9	2.2	11	2.7	9	2.2	5	1.2
7	Caused you to decrease your physical activities (exercising, sports, etc.)	368	91.5	19	4.7	4	1.0	7	1.7	3	0.7	1	0.2
8	Caused you to have problems with your partner or spouse	396	98.5	5	1.2	0	0.0	1	0.2	0	0.0	0	0.0
9	Made you uncomfortable while traveling with others because of needing to stop for a restroom	348	86.6	27	6.7	9	2.2	9	2.2	3	0.7	6	1.5
10	Affected your relationships with family and friends	383	95.3	11	2.7	7	1.7	1	0.2	0	0.0	0	0.0
11	Interfered with getting the amount of sleep you needed	361	89.8	26	6.5	4	1.0	8	2.0	2	0.5	1	0.2
12	Caused you embarrassment	344	85.6	27	6.7	11	2.7	12	3.0	6	1.5	2	0.5
13	Caused you to locate the closest restroom as soon as you arrive at a place you have never been	344	85.6	28	7.0	11	2.7	7	1.7	6	1.5	6	1.5

### Association of the sociodemographic, health, and academic characteristics with the OAB Bother Symptom and health related quality of life scores

There was a strong negative correlation between the transformed OAB symptom bother and HRQoL scores obtained in this study (Spearman’s rho = 64.4%, *p* value < 0.001).

The OAB symptom bother scores were significantly higher for students who studied dentistry, were of female gender, had chronic diseases, reported high stressful life, and were not satisfied with their social life compared to students who studied medicine, were male in gender, did not have chronic disease, reported less stressful life, and were satisfied with their social life. Comparison of the median and interquartile range of the OAB symptom bother scores is shown in Table 4. On the other hand, the HRQoL scores were significantly higher for students who studied medicine, were male in gender, reported low stressful life, studies less than 5 h, and were satisfied with their social life. Comparison of the median and interquartile range of the HRQoL scores is shown in Table 4.

**Table 4:** Comparison of the median and interquartile range of the overactive bladder symptom bother and health-related quality of life scores

Variable	n	%	OAB scores		HRQoL scores	
			Median [Q1, Q3]	*p* value	Median [Q1, Q3]	*p* value
University*						
University 1	250	62.2	54.1 [44.8, 81.9]	0.181	94.4 [90.4, 94.4]	0.362
University 2	114	28.4	54.1 [44.8, 91.1]		94.4 [86.5, 94.4]	
University 3	38	9.5	63.3 [44.8, 91.1]		94.4 [80.6, 94.4]	
Discipline						
Medicine	350	87.1	54.1 [44.8, 81.9]	0.003	94.4 [90.4, 94.4]	0.003
Dentistry	52	12.9	72.6 [44.8, 116.6]		93.4 [73.6, 94.4]	
Academic stage						
Basic stage (1st–3rd year)	197	49.0	54.1 [44.8, 81.9]	0.195	94.4 [88.4, 94.4]	0.329
Clinical stage (4th–6th year)	205	51.0	54.1 [35.6, 81.9]		94.4 [90.4, 94.4]	
Gender						
Male	126	31.3	54.1 [35.6, 72.6]	0.009	94.4 [94.4, 94.4]	0.001
Female	276	68.7	54.1 [44.8, 91.1]		94.4 [88.4, 94.4]	
Age (years)						
< 21	174	43.3	54.1 [44.8, 84.2]	0.213	94.4 [88.4, 94.4]	0.494
≥ 21	228	56.7	54.1 [44.8, 81.9]		94.4 [90.4, 94.4]	
Body mass index						
< 24.9	271	67.4	54.1 [44.8, 81.9]	0.994	94.4 [90.4, 94.4]	0.995
≥ 24.9	131	32.6	54.1 [44.8, 81.9]		94.4 [88.4, 94.4]	
Presence of chronic disease						
No	378	94.0	54.1 [44.8, 81.9]	0.018	94.4 [90.4, 94.4]	0.067
Yes	24	6.0	81.9 [44.8, 135.1]		94.4 [66.3, 94.4]	
History of surgery						
Yes	78	19.4	54.1 [44.8, 91.1]	0.531	94.4 [88.4, 94.4]	0.832
No	324	80.6	54.1 [44.8, 81.9]		94.4 [90.4, 94.4]	
History of trauma						
Yes	8	2.0	63.3 [44.8, 93.4]	0.666	92.4 [71.7, 94.4]	0.328
No	394	98.0	54.1 [44.8, 81.9]		94.4 [89.9, 94.4]	
Employment						
Yes	14	3.5	44.8 [35.6, 56.4]	0.092	94.4 [93.4, 94.4]	0.291
No	388	96.5	54.1 [44.8, 81.9]		94.4 [88.4, 94.4]	
Smoking						
Yes	65	16.2	63.3 [44.8, 91.1]	0.494	94.4 [88.4, 94.4]	0.758
No	337	83.8	54.1 [44.8, 81.9]		94.4 [89.4, 94.4]	
Self-rated satisfaction with academic achievement						
Not satisfied	82	20.4	54.1 [44.8, 81.9]	0.704	94.4 [89.9, 94.4]	0.866
Satisfied	320	79.6	54.1 [44.8, 81.9]		94.4 [88.4, 94.4]	
Self-rated stress						
Low stress	255	63.4	54.1 [35.6, 72.6]	0.003	94.4 [92.4, 94.4]	0.001
High stress	147	36.6	63.3 [44.8, 100.4]		94.4 [84.5, 94.4]	
Number of study hours/day						
< 5	177	44.0	54.1 [40.2, 81.9]	0.056	94.4 [92.4, 94.4]	0.028
≥ 5	225	56.0	54.1 [44.8, 91.1]		94.4 [88.4, 94.4]	
Self-rated satisfaction with the financial status						
Not satisfied	149	37.1	54.1 [44.8, 86.5]	0.259	94.4 [86.5, 94.4]	0.099
Satisfied	253	62.9	54.1 [44.8, 81.9]		94.4 [90.4, 94.4]	
Self-rated satisfaction with the social life						
Not satisfied	125	31.1	63.3 [44.8, 91.1]	0.009	94.4 [86.5, 94.4]	0.038
Satisfied	277	68.9	54.1 [44.8, 81.9]		94.4 [90.4, 94.4]	

*1, 2, and 3 denotes the largest universities in Palestine in terms of number of students, HRQoL: Health-related quality of life, Q1: Lower quartile, Q3: Upper quartile, OAB: Overactive bladder

The multiple linear regression model showed that OAB symptom bother scores were significantly higher for dentistry students, those of female gender, had chronic disease, and those reported stressful life. On the other hand, the model showed that HRQoL scores were significantly higher for medicine students, those who reported less stressful life, and those reported satisfaction with their social life. Details of the multiple linear analysis are shown in Table 5.

**Table 5:** Multiple linear regression between sociodemographic, health, and academic variables of the students with overactive bladder Symptom Bother health related quality of life scores

Variable	Unstandardized coefficients	SE	Standardized coefficients	t	*p* value
OAB symptom bother scores					
Discipline	16.35	6.05	0.13	2.70	0.007
Gender	8.88	4.41	0.10	2.01	0.045
Presence of chronic disease	18.92	8.55	0.11	2.21	0.028
Self-rated stress	11.50	4.26	0.13	2.70	0.007
Self-rated satisfaction with the social life	− 8.18	4.35	− 0.09	− 1.88	0.061
HRQoL scores					
Discipline	17.39	6.06	0.14	2.87	0.004
Gender	7.16	4.48	0.08	1.60	0.111
Self-rated stress	11.95	4.27	0.14	2.80	0.005
Number of study hours	4.88	4.14	0.06	1.18	0.240
Self-rated satisfaction with the social life	− 8.77	4.36	− 0.10	− 2.01	0.045

HRQoL: Health-related quality of life, OAB: Overactive bladder, SE: Standard error, t: t-statistics

Presence of any OAB symptom bother were associated with female gender (Pearson Chi-Square/Fisher's Exact Test = 5.20, *p* value = 0.027) and high self-reported stress (Pearson Chi-Square/Fisher's Exact Test = 4.91, *p* value = 0.032). Less than optimal HRQoL was associated with studying dentistry (Pearson Chi-Square/Fisher's Exact Test = 5.63, *p* value = 0.020), female gender (Pearson Chi-Square/Fisher's Exact Test = 12.17, *p* value < 0.001), high self-reported stress (Pearson Chi-Square/Fisher's Exact Test = 10.67, *p* value = 0.001), and studying ≥ 5 h/day (Pearson Chi-Square/Fisher's Exact Test = 4.89, *p* value = 0.028). Details of associations between presence of any OAB symptom bother and less than optimal HRQoL with sociodemographic, health, and academic variables of the students are shown in Table 6.

**Table 6:** Associations between presence of any overactive bladder symptom bother and less than optimal health-related quality of life with sociodemographic, health, and academic variables of the students

Variable	OAB symptoms						HRQoL					
	Absent		Present		χ^2^/FET	*p* value	Less than optimal		Optimal		χ^2^/FET	*p* value
	n	%	n	%			n	%	n	%		
University^*^												
University 1	58	14.4	192	47.8	1.27	0.553	88	21.9	162	40.3	1.89	0.388
University 2	23	5.7	91	22.6			37	9.2	77	19.2		
University 3	6	1.5	32	8.0			17	4.2	21	5.2		
Discipline												
Medicine	80	19.9	270	67.2	2.36	0.150	116	28.9	234	58.2	5.63	0.020
Dentistry	7	1.7	45	11.2			26	6.5	26	6.5		
Academic stage												
Basic stage (1st–3rd year)	35	8.7	162	40.3	3.42	0.070	76	18.9	121	30.1	1.79	0.210
Clinical stage (4th–6th year)	52	12.9	153	38.1			66	16.4	139	34.6		
Gender												
Male	36	9.0	90	22.4	5.20	0.027	29	7.2	97	24.1	12.17	< 0.001
Female	51	12.7	225	56.0			113	28.1	163	40.5		
Age (years)												
< 21	31	7.7	143	35.6	2.65	0.113	67	16.7	107	26.6	1.36	0.249
≥ 21	56	13.9	172	42.8			75	18.7	153	38.1		
Body mass index												
< 24.9	56	13.9	215	53.5	0.47	0.519	96	23.9	175	43.5	0.00	1.000
≥ 24.9	31	7.7	100	24.9			46	11.4	85	21.1		
Presence of chronic disease												
No	84	20.9	294	73.1	1.26	0.318	131	32.6	247	61.4	1.23	0.277
Yes	3	0.7	21	5.2			11	2.7	13	3.2		
History of surgery												
Yes	14	3.5	64	15.9	0.78	0.445	28	7.0	50	12.4	0.01	1.000
No	73	18.2	251	62.4			114	28.4	210	52.2		
History of trauma												
Yes	1	0.2	7	1.7	0.40	0.693	4	1.0	4	1.0	0.77	0.461
No	86	21.4	308	76.6			138	34.3	256	63.7		
Employment												
Yes	4	1.0	10	2.5	0.41	0.743	3	0.7	11	2.7	1.22	0.395
No	83	20.6	305	75.9			139	34.6	249	61.9		
Smoking												
Yes	15	3.7	50	12.4	0.09	0.869	21	5.2	44	10.9	0.31	0.671
No	72	17.9	265	65.9			121	30.1	216	53.7		
Self-rated satisfaction with academic achievement												
Not satisfied	19	4.7	63	15.7	0.14	0.764	29	7.2	53	13.2	0.00	1.000
Satisfied	68	16.9	252	62.7			113	28.1	207	51.5		
Self-rated stress												
Low stress	64	15.9	191	47.5	4.91	0.032	75	18.7	180	44.8	10.67	0.001
High stress	23	5.7	124	30.8			67	16.7	80	19.9		
Number of study hours/day												
< 5	44	10.9	133	33.1	1.93	0.181	52	12.9	125	31.1	4.89	0.028
≥ 5	43	10.7	182	45.3			90	22.4	135	33.6		
Self-rated satisfaction with the financial status												
Not satisfied	27	6.7	122	30.3	1.73	0.211	59	14.7	90	22.4	1.89	0.195
Satisfied	60	14.9	193	48.0			83	20.6	170	42.3		
Self-rated satisfaction with the social life												
Not satisfied	21	5.2	104	25.9	2.51	0.119	52	12.9	73	18.2	3.13	0.091
Satisfied	66	16.4	211	52.5			90	22.4	187	46.5		

HRQoL: Health-related quality of life, OAB: Overactive bladder, χ^2^/FET: Pearson Chi-Square/Fisher's Exact Test

Multivariate logistic regression showed that dentistry students were 1.94-fold (95% CI 1.05, 3.56) more likely to report less than optimal HRQoL compared to medicine students, female students were 1.91-fold (95% CI 1.16, 3.14) more likely to report less than optimal HRQoL compared to male students, and students who self-reported high stress were 1.88-fold (95% CI 1.21, 2.91) more likely to report less than optimal HRQoL compared to the students who self-reported low stress. Details of the multivariate logistic regression model are shown in Table 7.

**Table 7:** Multivariate regression analysis of association between overactive bladder symptom bother and health-related quality of life with sociodemographic, health, and academic variables of the students

Domain	Variable	β	SE	Wald	*p* value	OR	95% CI for OR	
							Lower	Upper
OAB symptom bother	Gender							
	Male	Reference						
	Female	− 0.49	0.25	3.66	0.056	0.61	0.37	1.01
	Self-rated stress							
	Low stress	Reference						
	High stress	− 0.51	0.27	3.52	0.061	0.60	0.35	1.02
	Constant	1.80	0.24	57.40	0.000	6.06		
HRQoL	Discipline							
	Medicine	Reference						
	Dentistry	0.66	0.31	4.50	0.034	1.94	1.05	3.56
	Gender							
	Male	Reference						
	Female	0.65	0.25	6.49	0.011	1.91	1.16	3.14
	Self-rated stress							
	Low stress	Reference						
	High stress	0.63	0.22	8.02	0.005	1.88	1.21	2.91
	Number of study hours/day							
	< 5	Reference						
	≥ 5	0.28	0.22	1.52	0.218	1.32	0.85	2.04
	Constant	− 0.65	0.33	3.89	0.048	0.52		

CI: Confidence interval, OR: Odds ratio, SE: Standard error

## Discussion

During their training, medical and dentistry students could be exposed to a huge amount of stress that deteriorates their HRQoL [15]. Identifying and addressing stressors are prerequisite steps in improving the HRQoL of medical and dentistry students. In this multicenter study, prevalence of OAB symptom bother among medical and dentistry students, the associated sociodemographic, health, and academic factors, and their impact on the HRQoL were identified. To the best of our knowledge, this is the first study to investigate prevalence of OAB symptom bother and HRQoL among medical and dentistry students. A considerable percentage of the students were bothered with OAB symptom bother which affected their HRQoL. The study showed strong negative association between OAB symptom bother and HRQoL scores. OAB symptom bother and HRQoL scores were associated with discipline and stressful life. Findings of this study could be informative to decision makers in academia, health regulatory authorities, and advocacy groups who could be interested in improving the HRQoL of medical and dentistry students.

Findings of this study showed considerable percentage of medical and dentistry students reporting some degree of OAB symptom bother which affected their HRQoL. Findings of this study were consistent with those reported among university students including those in healthcare disciplines like midwifery in the US, Turkey, and Slovak Republic [8, 13, 14]. Together, these findings might indicate higher prevalence of OAB symptom bother among students of stressful disciplines like dentistry and medicine and lower HRQoL compared to their age-matched peers in other disciplines. Several previous studies have reported high prevalence of anxiety and depressive symptoms among students in healthcare disciplines including medical students in Palestine [15, 18, 19, 22, 23]. Findings of this study showed that OAB symptom bother scores were higher among female students compared to male students. Although some studies reported that prevalence of OAB symptom bother among male and female patients was similar, the majority of the studies have reported higher prevalence of OAB symptom bother among females compared to males [7, 27]. Results of this study were consistent with those reported in the previous studies on the higher prevalence of OAB symptoms among female students compared to male students in Korea, Turkey, Slovak Republic, and Japan [7, 8, 14, 27–30]. Presence of chronic diseases was also associated with higher OAB symptom bother. Previous studies have shown that chronic diseases like diabetes, irritable bowel syndrome, asthma, heart disease, depression, arthritis, and neurological conditions like multiple sclerosis and cerebral palsy [31–34]. In a study among 1025 patients with type 2 diabetes, 14% reported OAB symptoms [35]. Matsumoto et al. reported that among patients with irritable bowel syndrome, about 33% reported concurrent OAB symptoms [36]. The study population in this study were young medical students in their twenties. However, chronic diseases were prevalent in a small percentage (6%) of them. Findings of this study might add to the ability of the OAB symptom bother scale used in this study to differentiate between patient populations [25]. Students who rated their life as stressful reported higher OAB symptoms and lower HRQoL scores compared to others who reported less stressful life. Findings of this study were consistent with those reported in previous studies in which perceive stress was significantly correlated with OAB symptoms and HRQoL [37–40]. Lai et al. showed that severity of OAB symptoms were associated with higher perceived psychological stress [38]. In another study among nurses, Zhang et al. showed that OAB symptoms were associated with occupational stress [39]. Findings of this study were not surprising as in our previous study, depressive and anxiety symptoms were highly prevalent among medical students in Palestine [19].

In all healthcare systems, medical and dentistry students are the future workforce of physicians and dentists in primary, secondary, and tertiary healthcare centers. Therefore, addressing their health issues and improving their well-being and HRQoL could be crucial for optimal delivery of healthcare services and sustainability of the healthcare system [41, 42]. Decision makers in academia, health regulatory authorities, and advocacy groups might need to address health issues among medical and dentistry students. Improving care of chronic diseases that might comorbid with OAB symptoms, reducing stress through implementation of mindfulness and/or lifestyle programs, and providing support to female students might reduce OAB symptoms and improve HRQoL among medical and dentistry students [41, 43, 44].

### Strengths and limitations

Findings of this study should be interpreted after considering a number of strengths and limitations. First, in this multicenter study, data were collected from the major universities in Palestine. This should have improved the external validity of the study. Second, although the scales used were previously validated, appropriate diagnostic tests were also used in this study to ensure test–retest reliability and internal consistency of the study tool. Third, the number of students included in this study was larger than the sample size needed. Additionally, the participants were diversified in terms of sociodemographic, health, and academic variables. The sample included participants from both genders, who rated their social and financial status differently. This should have added depth and width to the results obtained in this study. Fourth, associations between the OAB symptom bother and HRQoL scores with the sociodemographic, health, and academic variables of the students were investigated using powerful statistical tests.

This study had a number of limitations. First, the study was conducted among medical and dentistry students only. We did not compare the prevalence of OAB symptom bother among university students in nonmedical disciplines. It could have been interesting to compare findings of the medical and dentistry students with those in nonmedical disciplines. Future studies would be conducted to compare prevalence of OAB symptom bother and HRQoL among university students in different health and non-health related disciplines. Second, the study was conducted in a cross-sectional design. Findings of cross-sectional studies are limited to the time period in which the study was conducted. A longitudinal study could have shown more interesting results and might have allowed detecting and investigating changes in OAB and/or HRQoL scores. Third, the items in the study tool were in both English and Arabic. Forward and backward translations were used to ensure accuracy [45, 46]. The items originally in English were translated into Arabic which were then translated back into English. Previous Arabic tools used to measure OAB symptoms were also reviewed [47, 48]. It is noteworthy mentioning that English is the language of instructions and teaching in Palestinian medical and dentistry schools. Additionally, Test of English as a Foreign Language (TOEFL) is a prerequisite for medical education in Palestinian universities. Fourth, this study was an observational study using a questionnaire and the results obtained were self-reported. A clinical assessment supported by laboratory findings of the students who participated in the study should have permitted collecting more meaningful data. Comparing the scores obtained in this study with more objective data such as bladder diary should have allowed better interpretation of the findings. Fifth, we did not correlate OAB symptom bother and HRQoL scores with anxiety/stress scores that could have been measured using a validated tool. Investigating correlations between OAB symptom bother, HRQoL scores, and anxiety/stress scores could have provided more meaningful data. Finally, a convenience sampling technique was followed in this study to recruit the study participants. As the study was conducted during the ongoing COVID-19 pandemic, this sampling technique allowed collecting the number of participants needed for this study respecting the stay-at-home orders and keeping social distancing.

## Conclusions

Our findings suggest that OAB symptom bother were prevalent among medical and dentistry students across Palestinian universities. There was a strong negative association between OAB symptom bother and HRQoL scores. Decision makers in academia, healthcare authorities, and advocacy groups might need to design appropriate interventions to address health and wellbeing issues of medical and dentistry students. Using appropriate diagnostic procedures, reducing stress, and improving the social life might help in reducing the burden on OAB and improve the HRQoL of medical and dentistry students. More investigations should be conducted to investigate if such interventions are effective in reducing OAB symptoms and improving HRQoL of medical and dentistry students.

## Supplementary Information

**Additional file 1**.** Supplementary Table S1**. Adherence to STROBE Statement for reporting cross-sectional studies [1]. **Supplementary Table S2**. Comparison between the sociodemographic, health, and academic characteristics of the medical and dentistry students.

## Data Availability

All data relevant to this study were included in the manuscript or provided as supplementary materials. The datasets used and/or analyzed during the current study are available from the corresponding author on reasonable request.

## Abbreviations

CI: Confidence interval
HRQoL: Health-related quality of life
IRB: Institutional Review Board
MD: Doctor of Medicine
OAB: Overactive bladder
OAB-q SF: OAB symptom bother and health-related quality of life Short-Form
Q1: Lower quartile
Q3: Upper quartile
VIF: Variance inflation factor

## References

[1] Foley S, Choudhury N, Huang M, Stari A, Nazir J, Freeman R (2019). Quality of life in patients aged 65 years and older with overactive bladder treated with mirabegron across eight European countries: secondary analysis of BELIEVE. Int J Urol.

[2] Haylen BT, de Ridder D, Freeman RM, Swift SE, Berghmans B, Lee J, Monga A, Petri E, Rizk DE, Sand PK (2010). An International Urogynecological Association (IUGA)/International Continence Society (ICS) joint report on the terminology for female pelvic floor dysfunction. Int Urogynecol J.

[3] Coyne KS, Sexton CC, Vats V, Thompson C, Kopp ZS, Milsom I (2011). National community prevalence of overactive bladder in the United States stratified by sex and age. Urology.

[4] Irwin DE, Milsom I, Hunskaar S, Reilly K, Kopp Z, Herschorn S, Coyne K, Kelleher C, Hampel C, Artibani W (2006). Population-based survey of urinary incontinence, overactive bladder, and other lower urinary tract symptoms in five countries: results of the EPIC study. Eur Urol.

[5] Stewart W, Van Rooyen J, Cundiff G, Abrams P, Herzog A, Corey R, Hunt T, Wein A (2003). Prevalence and burden of overactive bladder in the United States. World J Urol.

[6] Milsom I, Stewart W, Thüroff J (2000). The prevalence of overactive bladder. Am J Manag Care.

[7] Eapen RS, Radomski SB (2016). Review of the epidemiology of overactive bladder. Res Rep Urol.

[8] Özgür Yeniel A, Mete Ergenoglu A, Meseri R, Hadimli A, Askar N, Mete Itil I (2012). The prevalence of probable overactive bladder, associated risk factors and its effect on quality of life among Turkish midwifery students. Eur J Obstet Gynecol Reprod Biol.

[9] Coyne KS, Payne C, Bhattacharyya SK, Revicki DA, Thompson C, Corey R, Hunt TL (2004). The impact of urinary urgency and frequency on health-related quality of life in overactive bladder: results from a national community survey. Value Health.

[10] Ahmad QT, Saffarini JH, Samara AM, Jabri DS, Safarini ZH, Banijaber YM, Jaradat A, Abushamma F, Zyoud SeH (2020). The impact of lower urinary tract symptoms on the quality of life during pregnancy: a cross-sectional study from Palestine. BMC Urol.

[11] Balzarro M, Rubilotta E, Mancini V, Trabacchin N, Oppezzi L, Li Marzi V, Fusco F, Serati M (2019). Impact of overactive bladder-wet syndrome on female sexual function: a systematic review and meta-analysis. Sex Med Rev.

[12] Xu D, Zhu S, Li H, Gao J, Mou H, Wang K (2019). Relationships among occupational stress, toileting behaviors, and overactive bladder in nurses: a multiple mediator model. J Adv Nurs.

[13] Reisch R, Rutt R, Dockter M, Sanders S (2018). Overactive bladder symptoms in female health profession students: bladder diary characteristics and impact of symptoms on health-related quality of life. J Women's Health (2002).

[14] Hagovska M, Švihra J, Buková A, Horbacz A, Dračková D, Švihrová V (2019). Comparison of body composition and overactive bladder symptoms in overweight female university students. Eur J Obstet Gynecol Reprod Biol.

[15] Kötter T, Wagner J, Brüheim L, Voltmer E (2017). Perceived Medical School stress of undergraduate medical students predicts academic performance: an observational study. BMC Med Educ.

[16] Angkurawaranon C, Jiraporncharoen W, Sachdev A, Wisetborisut A, Jangiam W, Uaphanthasath R (2016). Predictors of quality of life of medical students and a comparison with quality of life of adult health care workers in Thailand. Springerplus.

[17] Pagnin D, de Queiroz V (2015). Comparison of quality of life between medical students and young general populations. Educ Health (Abingdon).

[18] Malibary H, Zagzoog MM, Banjari MA, Bamashmous RO, Omer AR (2019). Quality of Life (QoL) among medical students in Saudi Arabia: a study using the WHOQOL-BREF instrument. BMC Med Educ.

[19] Shawahna R, Hattab S, Al-Shafei R (2020). Tab’ouni M: Prevalence and factors associated with depressive and anxiety symptoms among Palestinian medical students. BMC Psychiatry.

[20] Brenneisen Mayer F, Souza Santos I, Silveira PS, Itaqui Lopes MH, de Souza AR, Campos EP, de Abreu BA, Hoffman Ii I, Magalhães CR, Lima MC (2016). Factors associated to depression and anxiety in medical students: a multicenter study. BMC Med Educ.

[21] Bacchi S, Licinio J (2015). Qualitative Literature Review of the Prevalence of Depression in Medical Students Compared to Students in Non-medical Degrees. Acad Psychiatry.

[22] Ribeiro ÍJS, Pereira R, Freire IV, de Oliveira BG, Casotti CA, Boery EN (2018). Stress and quality of life among university students: a systematic literature review. Health Prof Educ.

[23] Almalki SA, Almojali AI, Alothman AS, Masuadi EM, Alaqeel MK (2017). Burnout and its association with extracurricular activities among medical students in Saudi Arabia. Int J Med Educ.

[24] Vandenbroucke JP, von Elm E, Altman DG, Gøtzsche PC, Mulrow CD, Pocock SJ, Poole C, Schlesselman JJ, Egger M (2007). Strengthening the reporting of observational studies in epidemiology (STROBE): explanation and elaboration. PLoS Med.

[25] Coyne KS, Thompson CL, Lai JS, Sexton CC (2015). An overactive bladder symptom and health-related quality of life short-form: validation of the OAB-q SF. Neurourol Urodyn.

[26] Coyne K, Revicki D, Hunt T, Corey R, Stewart W, Bentkover J, Kurth H, Abrams P (2002). Psychometric validation of an overactive bladder symptom and health-related quality of life questionnaire: the OAB-q. Qual Life Res.

[27] Sekido N, Hinotsu S, Kawai K, Shimazui T, Akaza H (2006). How many uncomplicated male and female overactive bladder patients reveal detrusor overactivity during urodynamic study?. Int J Urol.

[28] Ural ÜM, Gücük S, Ekici A, Topçuoğlu A (2021). Urinary incontinence in female university students. Int Urogynecol J.

[29] Kajiwara M, Inoue K, Mutaguchi K, Usui T (2006). The prevalence of overactive bladder and nocturnal enuresis in Japanese early adolescents: a questionnaire survey. Hinyokika kiyo Acta urologica Japonica.

[30] Shin DG, Kim HW, Lee JZ (2010). Effect of the amount of hours spent studying on the prevalence of overactive bladder in college women. Lower Urin Tract Symptoms.

[31] Liu R-T, Chung M-S, Lee W-C, Chang S-W, Huang S-T, Yang KD, Chancellor MB, Chuang Y-C (2011). Prevalence of overactive bladder and associated risk factors in 1359 patients with type 2 diabetes. Urology.

[32] Khavari R, Elias SN, Pande R, Wu KM, Boone TB, Karmonik C (2019). Higher neural correlates in patients with multiple sclerosis and neurogenic overactive bladder following treatment with intradetrusor injection of onabotulinumtoxinA. J Urol.

[33] Chen L-C, Kuo H-C (2019). Pathophysiology of refractory overactive bladder. LUTS Lower Urin Tract Symptoms.

[34] Kim KS, Kim H-J, Lee SH, Cho ST, Moon HS (2017). Association between irritable bowel syndrome and overactive bladder: a research survey. Urology.

[35] Xu D, Cheng R, Ma A, Zhao M, Wang K (2017). Toileting behaviors and overactive bladder in patients with type 2 diabetes: a cross-sectional study in China. BMC Urol.

[36] Matsumoto S, Hashizume K, Wada N, Hori J, Tamaki G, Kita M, Iwata T, Kakizaki H (2013). Relationship between overactive bladder and irritable bowel syndrome: a large-scale internet survey in Japan using the overactive bladder symptom score and Rome III criteria. BJU Int.

[37] Ge TJ, Vetter J, Lai HH (2017). Sleep disturbance and fatigue are associated with more severe urinary incontinence and overactive bladder symptoms. Urology.

[38] Lai H, Gardner V, Vetter J, Andriole GL (2015). Correlation between psychological stress levels and the severity of overactive bladder symptoms. BMC Urol.

[39] Zhang C, Hai T, Yu L, Liu S, Li Q, Zhang X, Xu T, Wang X (2013). Association between occupational stress and risk of overactive bladder and other lower urinary tract symptoms: a cross-sectional study of female nurses in China. Neurourol Urodyn.

[40] Chen G-D, Lin T-L, Hu S-W, Chen Y-C, Lin L-Y (2003). Prevalence and correlation of urinary incontinence and overactive bladder in Taiwanese women. Neurourol Urodyn.

[41] Hassed C, de Lisle S, Sullivan G, Pier C (2009). Enhancing the health of medical students: outcomes of an integrated mindfulness and lifestyle program. Adv Health Sci Educ.

[42] Henning MA, Krägeloh CU, Hawken SJ, Zhao Y, Doherty I (2012). The quality of life of medical students studying in New Zealand: a comparison with nonmedical students and a general population reference group. Teach Learn Med.

[43] Dyrbye L, Shanafelt T (2016). A narrative review on burnout experienced by medical students and residents. Med Educ.

[44] Moir F, Henning M, Hassed C, Moyes SA, Elley CR (2016). A peer-support and mindfulness program to improve the mental health of medical students. Teach Learn Med.

[45] Tsang S, Royse CF, Terkawi AS (2017). Guidelines for developing, translating, and validating a questionnaire in perioperative and pain medicine. Saudi J Anaesth.

[46] Boparai JK, Singh S, Kathuria P (2018). How to design and validate a questionnaire: a guide. Curr Clin Pharmacol.

[47] Al-Shaiji TF, Alkabbani M (2019). Validation of the Arabic linguistic version of the 8-item overactive bladder questionnaire (OAB-V8). Int Urogynecol J.

[48] Elbaset MA, Hashem A, Taha D-E, Zahran MH, El-Hefnawy AS (2019). Validation of the arabic linguistic version of the overactive bladder symptoms score questionnaire. Arab J Urol.

