# Supplementary material for: Prevalence of overactive bladder symptoms and their impact on health-related quality of life of medical and dentistry students: a multicenter cross-sectional study
Source: BMC Urol. 2021 Oct 8;21:142. doi: 10.1186/s12894-021-00909-1 (PMC8497683; doi:10.1186/s12894-021-00909-1)
Supplement: Supplementary file 1 — Additional file 1. Supplementary Table S1. Adherence to STROBE Statement for reporting cross-sectional studies [1]. Supplementary Table S2. Comparison between the sociodemographic, health, and academic characteristics of the medical and dentistry students. [file 12894_2021_909_MOESM1_ESM.docx]

**Supplementary materials for the manuscript**

**Prevalence of overactive bladder symptoms and their impact on health-related quality of life of medical and dentistry students: A multicenter cross-sectional study**

Ramzi Shawahna^1,2*^, Hatim Hijaz^3,4^, Khaled Jallad^3^, Mohammad Abushamma^3^, Mothana Sawafta^3^

^1^Department of Physiology, Pharmacology and Toxicology, Faculty of Medicine and Health Sciences, An-Najah National University, Nablus, Palestine

^2^An-Najah BioSciences Unit, Centre for Poisons Control, Chemical and Biological Analyses, An-Najah National University, Nablus, Palestine

^3^Department of Medicine, Faculty of Medicine and Health Sciences, An-Najah National University, Nablus, Palestine

^4^An-Najah National University Hospital, An-Najah National University, Nablus, Palestine

**^*^Correspondence:**

Ramzi Shawahna, PhD, Department of Physiology, Pharmacology and Toxicology, Faculty of Medicine & Health Sciences, New Campus, Building: 19, Office: 1340, An-Najah National University, P.O. Box 7, Nablus, Palestine

Phone: + (970) 923 45113 ext 2772

Phone: + (970) 92349739

Email: [ramzi_shawahna@hotmail.com](mailto:ramzi_shawahna@hotmail.com)

**Supplementary Table S1:** Adherence to STROBE Statement for reporting cross-sectional studies [[1](#_ENREF_1)]

| **Section** | **Item #** | **Recommendation** | **Place in the manuscript** |
| --- | --- | --- | --- |
| **Title and abstract** | 1 | (*a*) Indicate the study’s design with a commonly used term in the title or the abstract | The title and abstract indicate the study design. Please see the title page and the abstract. |
|  |  | (*b*) Provide in the abstract an informative and balanced summary of what was done and what was found | Please see the abstract. The abstract was formatted in compliance with the journal’s style |
| **Introduction** | | | |
| Background/rationale | 2 | Explain the scientific background and rationale for the investigation being reported | Introduction section: Paragraphs 3-5 |
| Objectives | 3 | State specific objectives, including any pre-specified hypotheses | Introduction section: the last paragraph |
| **Methods** | | | |
| Study design | 4 | Present key elements of study design early in the paper | Methods section: under Study design and the study tool |
| Setting | 5 | Describe the setting, locations, and relevant dates, including periods of recruitment, exposure, follow-up, and data collection | Methods section: under Study settings/context |
| Participants | 6 | (*a*) Give the eligibility criteria, and the sources and methods of selection of participants | Methods section: under Study population, sample size, and sampling |
| Variables | 7 | Clearly define all outcomes, exposures, predictors, potential confounders, and effect modifiers. Give diagnostic criteria, if applicable | Methods section: under Study design and the study tool |
| Data sources/ measurement | 8* | For each variable of interest, give sources of data and details of methods of assessment (measurement). Describe comparability of assessment methods if there is more than one group | Methods section: under Study design and the study tool |
| Bias | 9 | Describe any efforts to address potential sources of bias | Methods section: under Study design and the study tool and Pilot testing of the questionnaire |
| Study size | 10 | Explain how the study size was arrived at | Methods section: under Study population, sample size, and sampling |
| Quantitative variables | 11 | Explain how quantitative variables were handled in the analyses. If applicable, describe which groupings were chosen and why | Methods section: under Data analysis |
| Statistical methods | 12 | (*a*) Describe all statistical methods, including those used to control for confounding | Methods section: under Data analysis |
|  |  | (*b*) Describe any methods used to examine subgroups and interactions | Methods section: under Data analysis |
|  |  | (*c*) Explain how missing data were addressed | Not applicable |
|  |  | (*d*) If applicable, describe analytical methods taking account of sampling strategy | Not applicable |
|  |  | (*e*) Describe any sensitivity analyses | Not applicable |
| **Results** | | | |
| Participants | 13* | (a) Report numbers of individuals at each stage of study—eg numbers potentially eligible, examined for eligibility, confirmed eligible, included in the study, completing follow-up, and analysed | Results section: under Sociodemographic, health, and academic characteristics of the medical and dentistry students and Table 1. |
|  |  | (b) Give reasons for non-participation at each stage | Not applicable. |
|  |  | (c) Consider use of a flow diagram | Not applicable. |
| Descriptive data | 14* | (a) Give characteristics of study participants (eg demographic, clinical, social) and information on exposures and potential confounders | Results section: under Sociodemographic, health, and academic characteristics of the medical and dentistry students and Table 1. |
|  |  | (b) Indicate number of participants with missing data for each variable of interest | Not applicable. |
| Outcome data | 15* | Report numbers of outcome events or summary measures | Table 1-5 |
| Main results | 16 | (*a*) Give unadjusted estimates and, if applicable, confounder-adjusted estimates and their precision (eg, 95% confidence interval). Make clear which confounders were adjusted for and why they were included | Results section and Table 1-5 with the supplementary Tables. |
|  |  | (*b*) Report category boundaries when continuous variables were categorized | Results section and Table 1-5 with the supplementary Tables. |
|  |  | (*c*) If relevant, consider translating estimates of relative risk into absolute risk for a meaningful time period | Not applicable. |
| Other analyses | 17 | Report other analyses done—eg analyses of subgroups and interactions, and sensitivity analyses | Results section and Table 1-5 with the supplementary Tables. |
| **Discussion** | | | |
| Key results | 18 | Summarise key results with reference to study objectives | Discussion section: The first paragraph |
| Limitations | 19 | Discuss limitations of the study, taking into account sources of potential bias or imprecision. Discuss both direction and magnitude of any potential bias | Discussion section: under Strengths and limitations |
| Interpretation | 20 | Give a cautious overall interpretation of results considering objectives, limitations, multiplicity of analyses, results from similar studies, and other relevant evidence | Discussion section |
| Generalisability | 21 | Discuss the generalisability (external validity) of the study results | Discussion section: under Strengths and limitations |
| **Other information** | | | |
| Funding | 22 | Give the source of funding and the role of the funders for the present study and, if applicable, for the original study on which the present article is based | Not applicable. |

**Supplementary Table S2:** Comparison between the sociodemographic, health, and academic characteristics of the medical and dentistry students

|  | **Discipline** | | | |  |  |
| --- | --- | --- | --- | --- | --- | --- |
|  | **Medicine** | | **Dentistry** | |  |  |
| **Variable** | **n** | **%** | **n** | **%** | **Pearson Chi-Square/Fisher's Exact Test** | **p-value** |
| **Academic stage** |  |  |  |  |  |  |
| Basic stage (1^st^ - 3^rd^ year) | 167 | 47.7 | 30 | 57.7 | 1.80 | 0.185 |
| Clinical stage (4^th^ - 6^th^ year) | 183 | 52.3 | 22 | 42.3 |  |  |
| **Gender** |  |  |  |  |  |  |
| Male | 116 | 33.1 | 10 | 19.2 | 4.07 | 0.054 |
| Female | 234 | 66.9 | 42 | 80.8 |  |  |
| **Age (years)** |  |  |  |  |  |  |
| < 21 | 152 | 43.4 | 22 | 42.3 | 0.02 | 0.882 |
| ≥ 21 | 198 | 56.6 | 30 | 57.7 |  |  |
| **Body mass index** |  |  |  |  |  |  |
| < 24.9 | 231 | 66.0 | 40 | 76.9 | 2.46 | 0.153 |
| ≥ 24.9 | 119 | 34.0 | 12 | 23.1 |  |  |
| **Presence of chronic disease** |  |  |  |  |  |  |
| No | 333 | 95.1 | 45 | 86.5 | 4.73 | 0.057 |
| Yes | 17 | 4.9 | 7 | 13.5 |  |  |
| **History of surgery** |  |  |  |  |  |  |
| Yes | 70 | 20.0 | 8 | 15.4 | 0.62 | 0.463 |
| No | 280 | 80.0 | 44 | 84.6 |  |  |
| **History of trauma** |  |  |  |  |  |  |
| Yes | 6 | 1.7 | 2 | 3.8 | 0.87 | 0.604 |
| No | 344 | 98.3 | 50 | 96.2 |  |  |
| **Employment** |  |  |  |  |  |  |
| Yes | 12 | 3.4 | 2 | 3.8 | 0.02 | 1.000 |
| No | 338 | 96.6 | 50 | 96.2 |  |  |
| **Smoking** |  |  |  |  |  |  |
| Yes | 51 | 14.6 | 14 | 26.9 | 5.10 | 0.028 |
| No | 299 | 85.4 | 38 | 73.1 |  |  |
| **Self-rated satisfaction with academic achievement** |  |  |  |  |  |  |
| Not satisfied | 75 | 21.4 | 7 | 13.5 | 1.77 | 0.202 |
| Satisfied | 275 | 78.6 | 45 | 86.5 |  |  |
| **Self-rated stress** |  |  |  |  |  |  |
| Low stress | 218 | 62.3 | 37 | 71.2 | 1.54 | 0.223 |
| High stress | 132 | 37.7 | 15 | 28.8 |  |  |
| **Number of study hours/day** |  |  |  |  |  |  |
| < 5 | 162 | 46.3 | 15 | 28.8 | 5.59 | 0.024 |
| ≥ 5 | 188 | 53.7 | 37 | 71.2 |  |  |
| **Self-rated satisfaction with the financial status** |  |  |  |  |  |  |
| Not satisfied | 131 | 37.4 | 18 | 34.6 | 0.15 | 0.760 |
| Satisfied | 219 | 62.6 | 34 | 65.4 |  |  |
| **Self-rated satisfaction with the social life** |  |  |  |  |  |  |
| Not satisfied | 108 | 30.9 | 17 | 32.7 | 0.07 | 0.873 |
| Satisfied | 242 | 69.1 | 35 | 67.3 |  |  |

**Reference**

1. Vandenbroucke JP, von Elm E, Altman DG, Gotzsche PC, Mulrow CD, Pocock SJ, Poole C, Schlesselman JJ, Egger M: **Strengthening the Reporting of Observational Studies in Epidemiology (STROBE): explanation and elaboration**. *PLoS medicine* 2007, **4**(10):e297.
